# Modeling social support and life satisfaction in running groups through stress management

**DOI:** 10.1186/s12889-026-26378-5

**Published:** 2026-01-26

**Authors:** Ezgi Kurşun, Junhyoung Kim, Hüseyin Gümüş, Nezaket Bilge Uzun

**Affiliations:** 1 Department of Sport Sciences, Mersin University, Mersin, Turkey; 2 Department of Health Behaviors, School of Public Health, Texas A&M University, College Station, Texas USA; 3 Department of Education, Mersin University, Mersin, Turkey

**Keywords:** Recreational running, Leisure-time physical activity, Mediation

## Abstract

**Background:**

The fast-paced and stressful conditions of modern life have increased the need for social and emotional resources that enhance individuals’ psychological resilience. Leisure activities, especially group-based physical exercises, offer significant social and psychological benefits. Among these, recreational running groups stand out, positively influencing participants in various ways. This study aimed to examine the mediating role of stress management in the relationship between recreational runners’ perceived social support and life satisfaction using structural equation modeling analysis.

**Methods:**

A predictive correlational survey model was employed. Data were collected using the Leisure Time Stress Coping Strategies Scale, the Multidimensional Perceived Social Support Scale, and the Life Satisfaction Scale. The study included 361 recreational athletes (147 women, 214 men) participating in running events organized in Mersin and its surroundings. Prior to analysis, z-values and Mahalanobis distances were calculated to assess normality, and VIF and tolerance values were examined to check for multicollinearity. The measurement model was tested via confirmatory factor analysis, and goodness-of-fit values were evaluated. Structural relationships between variables were then examined using structural equation modeling.

**Results:**

Social support significantly predicted life satisfaction (β = 0.37). When stress management was included, the predictive effect of social support on life satisfaction decreased (β = 0.33) but remained significant. Social support also significantly predicted stress management (β = 0.26). These results indicate that stress management partially mediates the relationship between social support and life satisfaction.

**Conclusion:**

Perceived social support and effective stress management strategies are important factors in enhancing life satisfaction among recreational runners. These findings highlight the role of social and psychological resources in promoting well-being in leisure contexts.

## Introduction

**Fig. 1:**
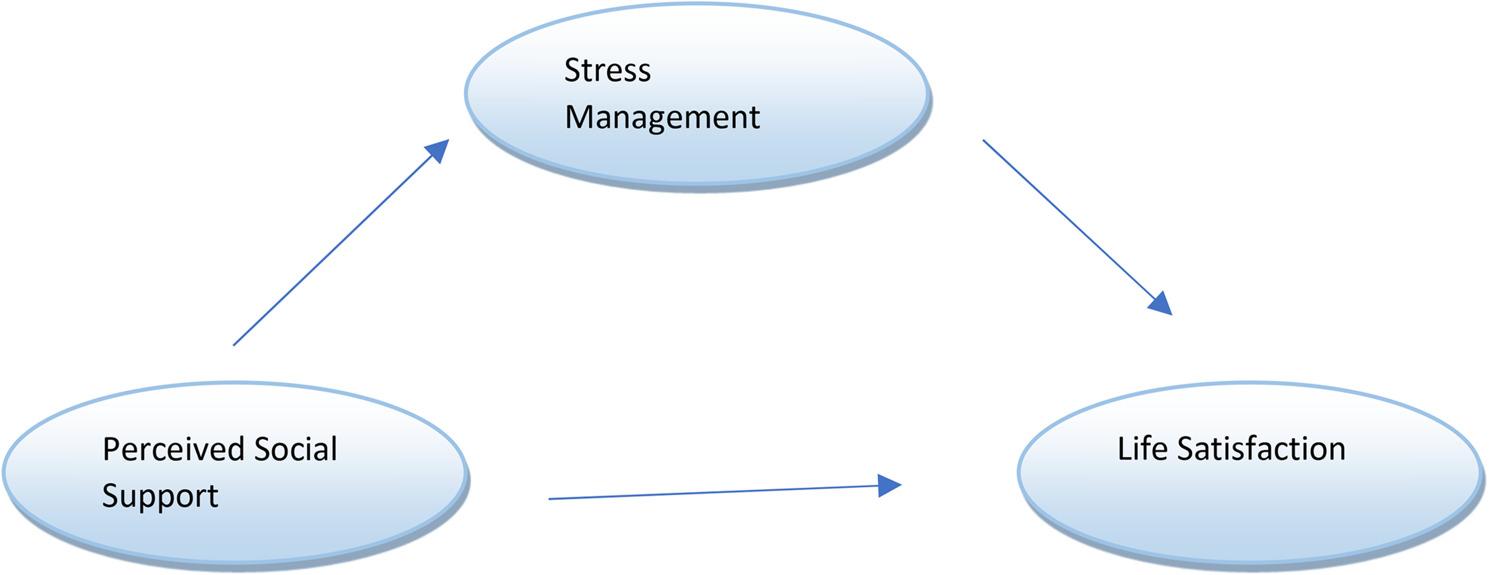
Theoretical model to be tested and associated hypotheses (H1-H4)

 Modern life has brought about rapid changes across economic, social, and technological domains. Increased urbanization, demanding work schedules, and the pressure to keep pace with fast-moving lifestyles have introduced new sources of stress. Individuals develop various strategies to cope with these challenges and the resources and coping approaches they adopt play a fundamental role in shaping overall quality of life [1]. These strategies are also effective in managing the sources of stress [2]. leisure time—defined as free time outside obligatory work and life responsibilities-represents an important stress-coping resource that supports stress management [3]. Accordingly, placing sufficient emphasis on leisure-time activities plays a vital role in preventing or alleviating potential psychological and social problems [4]. When individuals participate in preferred leisure activities, they not only experience enjoyment and psychological detachment from daily demands, but also develop social identity through interaction with others-demonstrating the meaningful role of leisure in human life [5–7]. Among leisure activities, physical activity is particularly common as a means of promoting more active lifestyles in communities [8]. One of the most prominent among such activities in recent years is recreational running, which has gained increasing global attention [9]. Recreational running is accessible, requires minimal equipment, and is easily practiced activity enjoyed by individuals from all segments of society, regardless of class distinctions, and is often pursued in one’s leisure time for fun and stress relief [10–12]. Recreational runners often experience both physical and psychological challenges, particularly during long-distance efforts [13, 14]. In the regulation of stress through internal dialogue during running contributes to a state of psychological well-being and facilitates the experience of flow throughout the activity. In a study with recreational runners, participants reported that engaging in recreation made them feel better, and many described the activity as a form of therapy [15]. Similar findings have been reported in studies examining individuals who participate in leisure-time sports, suggesting that engaging in stress-coping behaviors through leisure activities contributes meaningfully to psychological well-being [16]. In this context, social support is emphasized as a significant factor in participation in physical activity. Leisure-time physical activities, such as recreational running, are considered important sources of social support, and perceived social support during leisure time has been shown to have stress-reducing effects [17, 18]. Furthermore, as stress decreases, improvements in quality of life may contribute to higher life satisfaction. Previous studies have highlighted that quality of life is an important parameter in assessing life satisfaction [19]. Additionally, research has shown that participation in social activities during leisure time reduces feelings of loneliness and social isolation, while simultaneously enhancing perceived social support [20]. In line with this, the present study investigates the mediating role of stress management in the relationship between perceived social support—considered as the independent variable—and life satisfaction—considered as the dependent variable. In mediation studies, mediating variables are defined as variables that transmit the effect of the independent variable to the dependent variable in the relationship between the two. Assessing mediating mechanisms reveals the impact of an intervention on the relationship between the independent and dependent variables [21]. Thus, the presence of a mediation effect indicates that variations in the mediator also influence the strength of the relationship [22]. Additionally, psychological conditions such as perceived social support and life satisfaction, which are examined within the scope of this research, may also be influenced by individual, environmental, and social factors. Indeed, previous studies have shown that variables like happiness, life satisfaction, stress, and psychological well-being are shaped by various environmental and behavioral influences [13, 16, 17, 24]. In this context, leisure itself is a dynamic construct that can take different forms depending on political, social, and economic conditions. Given this characteristic, it is suggested that an individual’s ability to manage stress may play a determining role in the relationship between perceived social support and life satisfaction. Accordingly, stress management is considered a mediating variable that could influence this relationship. Proposing a model to explain how these relationships are formed represents a relatively underexplored area in the existing literature. Recreational running, in particular, is characterized as an unstructured activity that nonetheless fosters a high level of social interaction. The present study aims to contribute to a deeper understanding of the relationship between perceived social support and life satisfaction by highlighting the mediating role of stress management. This research is significant in that it offers a unique perspective to the literature on both leisure-time psychology and community-based physical activity studies. Accordingly, the aim of this study is to examine the relationship between perceived social support and life satisfaction among members of running groups, as well as the potential mediating role of stress management, within the framework of the theoretical model presented in Fig. 1.

Based on the directional relationships illustrated in Figure 1, the hypotheses of the study are formulated as follows:

- H1: There is a significant positive relationship between perceived social support and life satisfaction.
- H2: There is a significant positive relationship between perceived social support and stress management.
- H3: There is a significant positive relationship between stress management and life satisfaction.
- H4: Stress management plays a mediating role in the relationship between perceived social support and life satisfaction.

## Method

### Research model

This study employs the predictive correlational research design, which is a subtype of relational survey models. Predictive correlational research not only investigates the relationships between variables but also aims to make predictions based on the identified relationships [25]. The statistical analysis method used in this study is Structural Equation Modeling (SEM), which, while inherently relational, also enables the exploration of causal relationship patterns within a theoretical framework [26]. Within this context, the study investigates the mediating role of stress management in the relationship between perceived social support and life satisfaction.

### Study group

The sample of this study consisted of individuals who participated in recreational running events held in Mersin and its districts between 2024 and 2025. Mersin was selected as the data collection site due to its regular running events, its high level of participant turnout, and its accessibility to the recreational running community. Although the events were held in Mersin, runners and running groups from other regions of Türkiye also participated. Therefore, despite the geographically localized sampling frame, the sample represents a broader recreational runner population.The Demographic characteristics of participants are summarized in Table 1.

**Table 1:** Demographic characteristics of participants

Variables	Groups	F	%
Gender	Female	147	40,7
	Male	214	59,3
Frequency of Sport Participation	Once a week or less	132	36,6
	2–4 times a week	170	47,1
	5 times a week or more	59	16,3
Mode of Sports Participation	In groups	196	54,4
	Individually	164	45,6

As seen in Table 1, the participants exhibit a heterogeneous distribution across various demographic characteristics.

### Data collection tools

Within the scope of this study, evidence for the reliability and validity of the proposed model was also obtained from the collected data set. The values related to these evidences are presented in Table 2.

**Table 2:** Validity and reliability evidence for the scales used in the study

Meausrement Instruments	CR	AVE	MSV	MSA
Leisure Time Stress Coping Strategies	0.94	0.85	0.13	0.89
Life Satisfaction	0.91	0.68	0.13	0.76
Perceived Social Support	0.93	0.81	0.13	0.73
	CR > 0.70 CR > AVE	AVE > 0.50	MSV < AVE	MSA > 0.50

*CR* (Composite Reliability), *AVE* (Average Variance Extracted), *MSV *(Maximum Shared Variance), *MSA *(Measurement Sampling Adequacy)

####  Leisure-based stress coping strategies scale

The original scale consisted of 18 items in its first development [27]. In the Turkish adaptation study, a translation and back-translation procedure was applied to ensure linguistic equivalence, followed by factor analysis to examine construct validity. As a result, three items were removed, and the final version included 15 items. The adapted scale retained three subdimensions: Leisure Companionship, Temporary Coping, and Mood Improvement [28]. The Cronbach’s alpha reliability coefficients were reported as 0.87, 0.88, and 0.84 for the subdimensions, and 0.93 for the overall scale.

#### Satisfaction with life scale

The scale was originally developed as a unidimensional structure consisting of 5 items [29]. In the Turkish adaptation, a translation and back-translation procedure was performed to ensure linguistic equivalence, followed by expert review for content validity. To evaluate the temporal stability of the instrument, a test–retest reliability study was conducted, demonstrating a strong correlation between the two administrations. The adapted version retained its single-factor structure [30]. The Cronbach’s alpha reliability coefficient was calculated as 0.88, and the total explained variance was reported as 68.3%.

#### Multidimensional scale of perceived social support

The original scale consists of 12 items structured under three subdimensions [31]. In the Turkish adaptation, a translation and back-translation procedure was applied, linguistic equivalence was established, and the three-factor structure was retained.Reliability analyses in the adaptation study demonstrated high internal consistency values for both total and subscale scores [32]. In the present study, Cronbach’s alpha coefficients were calculated as 0.92 for the significant other subdimension, 0.85 for the family subdimension, 0.88 for the friends subdimension, and 0.89 for the overall scale.

Within the scope of this study, the model fit of the scales used was examined through Confirmatory Factor Analysis (CFA). The reliability and validity evidence obtained are presented in Table 2 below. For the first time in this study, the psychometric properties of the measurement tools were assessed using factor analytic theory applied to the research data, including McDonald’s omega (CR) reliability coefficients, average variance extracted (AVE) for convergent validity, as well as maximum shared variance (MSV) and average shared variance (ASV) for discriminant validity. These values allow for an evaluation of the reliability and validity of the model established in this research.The validity and reliability evidence for measurement tools are presented in Table 2.

When examining the values obtained for reliability and validity in Table 2, it was concluded that all items included in the scales adequately represent the constructs being measured. Additionally, the measurements within the model are reliable, and the relationships among the measurement instruments used in the study are strong.

### Data analyses

The study titled “Modeling Stress Management and Life Satisfaction in Running Groups through Stress Management Mediation” was designed to test a theoretical model. Accordingly, this model testing was carried out using Structural Equation Modeling (SEM). SEM investigates the relationships among latent variables involved in the study. The tested model is fundamentally examined through a combination of factor analysis and regression analysis [33]. Analyses conducted on latent variables within the model play a crucial role in revealing the true relationships obtained. Therefore, the relationships derived from SEM analyses are also known as true relationships [34]. Consequently, as stated in the research type, causal relationships are theoretically established.

SEM is a multivariate analysis method that requires testing certain assumptions before starting the analyses, such as sample size, missing data, multivariate normality, linearity, and multicollinearity issues. Regarding sample size, a sample size of 50 is considered very poor, 100 poor, 300 good, and 500 very good [35]. The minimum sample size should be 300 [36]. Considering these criteria, the sample size in the current study was deemed sufficient. The data were examined for missing values, and no missing data were found. Outliers were assessed by calculating Z-scores and Mahalanobis distances. In studies with sample sizes over 100, Z-scores between + 4 and − 4 are acceptable [37].To detect univariate outliers, Z-scores were examined, and all values were found to range between 1.8 and − 3.69, indicating no univariate outliers. For multivariate outliers, observations exceeding the chi-square critical value of χ²(26, 0.001) = 16.81 were considered multivariate outliers; thus, 13 observations were excluded from the analysis. Subsequently, skewness and kurtosis values were examined to test the normality assumption, with the relevant values presented in Table 3.

**Table 3:** Normality test results for the Leisure-Time stress coping strategies Scale, perceived social support Scale, and life satisfaction Scale; Means, standard Deviations, Skewness, and kurtosis values

Scale	Sub-Dimension	Min.	Max.	‾X	sd	Skewness	Kurtosis
Leisure Time Stress Coping Strategies Scale	Leisure Companionship	5.00	35.00	26.64	6.72	-1.14	0.97
	Temporary Coping	6.00	42.00	31.75	7.77	-1.17	1.21
	Mood Improvement	4.00	28.00	22.46	5.43	-1.48	2.20
	Total	15.00	105.00	80.85	18.86	-1.34	1.67
Multidimensional Perceived Social Support Scale	Family	4.00	28.00	21.94	5.95	-1.12	0.58
	Significant Other	4.00	28.00	22.26	5.35	-1.17	1.02
	Friends	4.00	28.00	22.05	5.40	-1.19	1.32
	Total	12.00	84.00	66.25	15.61	-1.17	1.13
Life Satisfaction Scale		5.00	25.00	15.36	4.54	-0.05	-0.14

In Table 3, skewness and kurtosis values were examined to determine whether the data collected within the scope of the current study follow a normal distribution. These values should lie within the range of -1 to + 1 [25]. Skewness and kurtosis values between − 1.5 and + 1.5 are also considered acceptable [36]. Values within the range of -2 to + 2 are acceptable [38], whereas values between − 3 and + 3 meet the normality criteria [39]. Examining the values in the table, all fall within the − 3 to + 3 range, indicating that the parameters of normal distribution are met.For multicollinearity assessment, Variance Inflation Factor (VIF) and Tolerance values were examined; all VIF values were below 5 and tolerance values above 0.20, indicating no multicollinearity problem.

In this study, mediation analyses were conducted following the mediation procedures proposed by Baron and Kenny [23]. Four steps are outlined as necessary to establish a mediation model.The independent variable must have a significant effect on the dependent variable.

1. The independent variable must have a significant effect on the mediator variable.
2. When controlling for the independent variable, the mediator must significantly predict the dependent variable.
3. When the mediator’s effect is controlled, there should be a significant reduction or elimination of the relationship between the independent and dependent variables. If the independent variable’s effect disappears completely, this indicates full mediation.

According to Baron and Kenny’s approach, the conditions for establishing mediation must be met sequentially. Full mediation occurs when the entire effect is transmitted through the mediator variable. In cases of full mediation, there is no direct effect between the independent and dependent variables; the effect of the independent variable on the dependent variable is entirely explained by the mediator variable. Therefore, the total effect equals the indirect effect [23].

In partial mediation, there remains a relationship between the independent and dependent variables that is not fully explained by the mediator [40]. Thus, when the mediator is included in the model, the direct effect of the independent variable on the dependent variable does not reduce to zero but is smaller than the total effect. This condition is referred to as partial mediation [23].

**Fig. 2:**
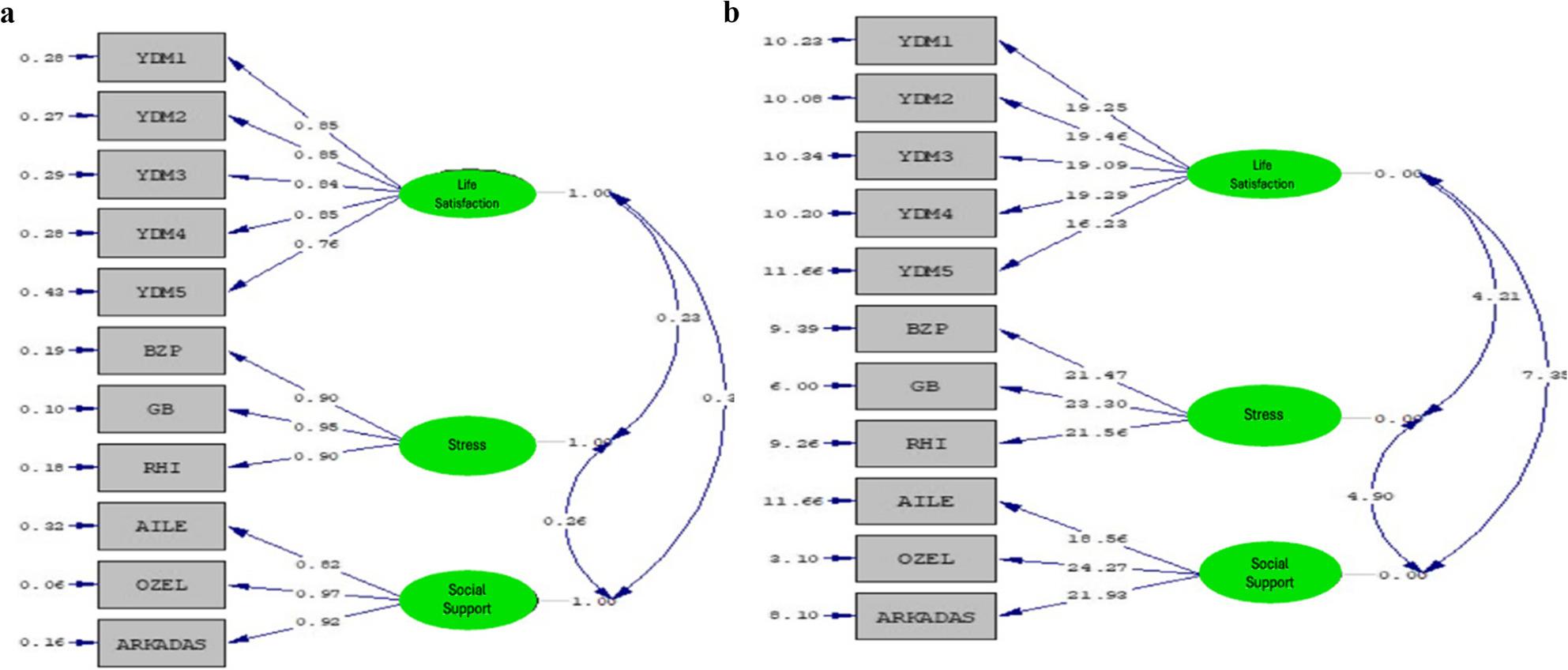
**a**. Standardized coefficients of the measurement model. **b**. t values related to the coefficients measurement model

Before proceeding to mediation analyses, two fundamental conditions need to be checked. One of these is that the measurement model for the variables used in the study and forming the structural model is well-fitting. In this context, the findings of the Confirmatory Factor Analysis (CFA) related to the measurement model are presented in Table 4 below. The following Fig. 2a. and 2b. present the standardized coefficients and t-values for this measurement model.

**Table 4:** Fit indices of the measurement model

Measurement Model	ꭓ^2^/df	CFI	RMSEA	NNFI	SRMR
Obtained Value	1,5	0.99	0.041	0.99	0.032
Acceptable Fit	2.00–3.00	0.90–0.94	0.05–0.08	0.90–0.94	0.05–0.08
Excellent Fit	0.00–2.00	0.95-1.00	0.00-0.05	0.95-1.00	0.00-0.05

When examining the model presented in Fig. 1a, the standardized coefficients for the Life Satisfaction Scale range between 0.85 and 0.76. For the Leisure Coping Strategies Scale, the coefficients range from 0.90 to 0.95. Regarding the Perceived Social Support Scale, the standardized values vary between 0.97 and 0.82. Figure 1b presents the t-values corresponding to the relationship coefficients. For the coefficients to be considered statistically significant, their t-values must fall outside the ± 1.96 range [36]. All values shown in the figure exceed this threshold, indicating that the corresponding coefficients are statistically significant.

**Table 5:** Bivariate correlations between variables

Variable	Life Satisfaction	Perceived Social Support	Stress Management
Life Satisfaction		r: 0.35 ***p*** **< 0.01**	r: 0.20 ***p*** **< 0.01**
Perceived Social Support			r: 0.23 ***p*** **< 0.01**
Stress Management			

Bold values indicate statistical significance at *p* < 0.01

The model fit indices recommended by Tabachnick and Fidell (2019) are presented in Table 3. The CFI and NNFI values exceeding 0.95, along with SRMR and RMSEA values being very close to zero, indicate an excellent fit of the model. The figure below presents the standardized coefficients and t-values related to this measurement model. The second aspect that needs to be checked involves the bivariate relationships among the variables in the model, which are presented in Table 5.

Table 5 examines the bivariate relationships among the variables. A moderate positive correlation was found between life satisfaction and perceived social support (*r* = 0.35, *p* < 0.01). Additionally, a weak positive relationship was observed between life satisfaction and stress management (*r* = 0.20, *p* < 0.01), as well as between perceived social support and stress management (*r* = 0.23, *p* < 0.01).

## Results

**Fig. 3:**
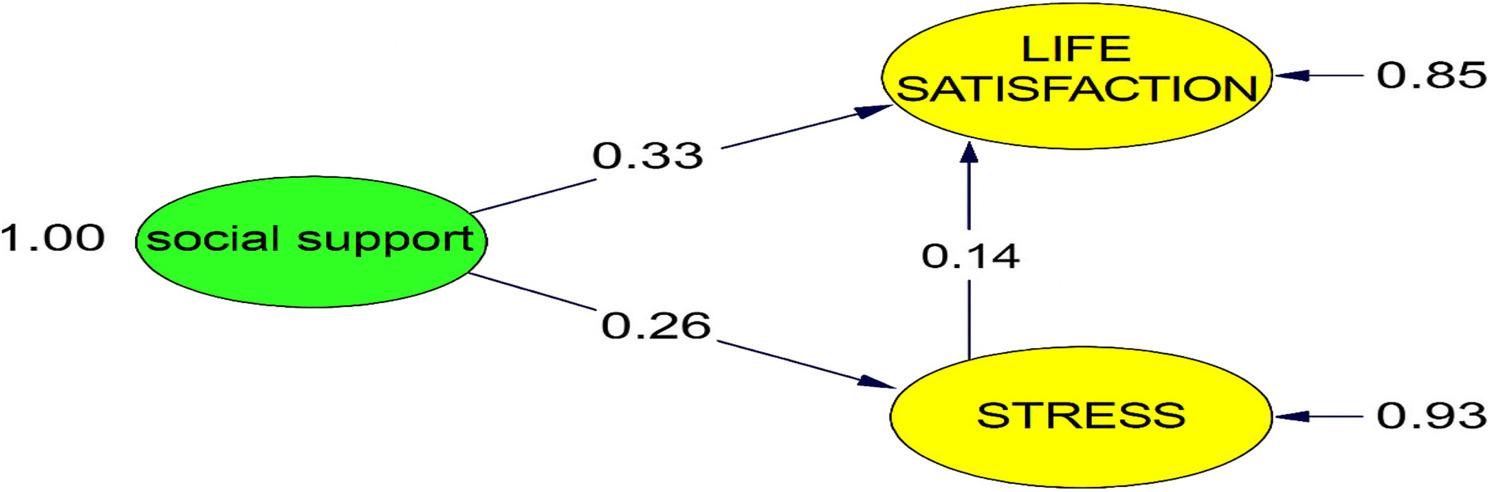
Path diagram of the SEM analysis examining the mediating role of stress management in the relationship between perceived social support and life satisfaction

After examining the fit of the measurement model and the bivariate relationships among the variables prior to the SEM analysis, the tested structural model investigating the mediating role of stress management in the relationship between perceived social support and life satisfaction is presented in Fig. 3. The hypotheses tested and the types of effects identified after all analyses are presented in Table 6.

When examining the path diagram of the SEM analysis presented in Fig. 3, the direct relationship between perceived social support and life satisfaction decreased from β = 0.37 (*p* < 0.001) to β = 0.33 (*p* < 0.001) after including stress management as a mediating variable, while the effect remained significant. These effects were further supported by the 95% confidence intervals estimated via bootstrap, as shown in Table 6.

**Table 6:** Bootstrap estimates of direct, indirect, and total effects of the mediation model (95% CI)

		%95 Confidence Interval			
Effect	SE	Lower	Upper	Z	*P*
Indirect	0.0183	0.008	0.0817	2.19	.**029**
Direct	0.0532	0.058	0.271	3.01	**0.003**
Total	0.0503	0.104	0.307	3.97	**0.001**

Bold values indicate statistical significance at *p* < 0.01

The bootstrap mediation analysis presented in Table 6 shows the effects of perceived social support (X) on life satisfaction (Y) through stress management (M). The indirect effect was significant (β = 0.0183, SE = 0.008, 95% CI [0.008, 0.082], Z = 2.19, *p* = 0.029), as was the direct effect (β = 0.0532, SE = 0.058, 95% CI [0.058, 0.271], Z = 3.01, *p* = 0.003), and the total effect (β = 0.0503, SE = 0.104, 95% CI [0.105, 0.307], Z = 3.97, *p* = 0.001). These results support a partial mediation model, confirming that stress management partially mediates the relationship between perceived social support and life satisfaction.

This indicates that stress management partially mediates this relationship. The hypotheses tested and the types of effects identified after all analyses are presented in Table 6.

Table 7. shows that the positive and significant effects between the paired variables specified in the study’s hypotheses, as well as the mediation effect, were confirmed, and all the hypotheses proposed within the scope of the research were accepted.

**Table 7:** Hypotheses and types of effects related to the research mode

Hypotheses	Path	Standardized Coefficient (β)	Effect Type	*p*	Result
H1	Social Support → Life Satisfaction	0.37	Total Effect	*P* < 0.01	Accepted
H2	Social Support → Stress Management	0.26	Effect to Mediator	*P* < 0.01	Accepted
H3	Stress Management → Life Satisfaction	0.23	Effect From Mediator	*P* < 0.01	Accepted
H4	Social Support → Stress Management → Life Satisfaction	0.33	Indirect Effect	*P* < 0.01	Accepted

## Discussion

This study examined the mediating role of stress management on the relationship between perceived social support and the life satisfaction of recreational runners using Structural Equation Modeling (SEM). SEM revealed the predictive relationships between social support, life satisfaction, and stress management, demonstrated that social support enhances life satisfaction through stress management, and provided evidence of a partial mediation effect of stress coping strategies in this relationship.

Positive correlations were found among all study variables. The first relationship identified was the positive association between perceived social support and stress management. In other words, as perceived social support increases, so does effective stress management. This finding can be interpreted as the perception that needed social support, when available, plays a constructive role in managing and regulating stress. Additionally, the presence of social support can enhance the psychological resilience and stress coping strategies that emppower individuals to become more resistant to adversity. Supporting this, the findings of previous research have suggested that stress management is an interactive process nourished by social resources [41]. Research findings have also provided evidence that engaging in sports activities during leisure time creates a source of social support that has a stress-reducing effect [18], and that adults with higher levels of perceived social support tend to use more effective methods for coping with stress [42]. Another study produced evidence of a positive association between perceived social support and life satisfaction (CITATION). This finding indicates that increased social support corresponds with higher levels of life satisfaction. It can be argued that perceived social support acts as a buffer against stressful situations and enhances life satisfaction. During recreational runs, cheering and supporting one another towards common running goals likely reinforces the perception of social support that contributes to positive psychological outcomes. Supporting this notion, research has indicated that social networks established in recreational runs and the collective participation in this activity increase engagement and highlight the enjoyment derived from the event [43]. The unique social aspect of running events may therefore positively affect life satisfaction. Consistent with this, university students with high levels of perceived social support also exhibited higher level of life satisfaction [44]. One study examining life satisfaction and social support identified positive interpersonal interactions and friendships as the strongest predictors of life satisfaction, mediated through perceived social support [45].

Finally, our findings indicated that stress management mediates the relationship between perceived social support and life satisfaction. This suggests that leisure activities, being self-directed and intrinsically motivated experiences, positively influence life satisfaction. Therefore, higher levels of leisure activity participation can be expected to enhance life satisfaction. The national and international literature revealed that participants in recreational running events reported feeling better and deriving therapeutic effects from participation in these activities, while others noted that the individuals engagig in such activities have fun and relieve stress [12, 16, 46, 47].

## Conclusion

This study examined the mediating role of leisure-based stress coping strategies in the relationship between perceived social support and life satisfaction among recreational runners. The findings indicate a positive association between perceived social support and life satisfaction, and reveal that stress coping strategies developed through leisure activities partially mediate this relationship. In particular, participation in recreational running as a form of leisure activity appears to support the development of more effective stress coping skills, thereby enhancing participants’ life satisfaction. These results suggest that leisure activities should be viewed not only as contexts for physical activity, but also as meaningful social environments in which psychological coping resources are developed.

From a theoretical perspective, the present study contributes to the leisure and public health literature by integrating perceived social support, leisure-based stress coping strategies, and life satisfaction within a single explanatory model. Conceptualizing leisure-based stress coping strategies as a mediating variable extends previous research in which these constructs have often been examined in isolation. From a practical perspective, the findings indicate that leisure activity programs should be designed not only to promote physical activity, but also to support conscious stress management and social interaction. Recreational running programs structured in this manner may serve as a sustainable and effective tool for enhancing participants’ psychological well-being and life satisfaction.

Future research should consider different types of recreational activities to emphasize the importance of activity type. Using various moderating variables may yield more comprehensive results. This study has some limitations. It is limited to recreational runners participating in running events held in Mersin and surrounding provinces during 2024–2025.

## Data Availability

The datasets used and/or analyzed during the current study are available from the corresponding author on reasonable request.
